# THE EQUIVALENCE OF CANTAB AS A COGNITIVE FUNCTION MEASURE FOR CHINESE STROKE SURVIVORS: A PILOT STUDY

**DOI:** 10.1093/geroni/igac059.2268

**Published:** 2022-12-20

**Authors:** Fanfan Li, Huanzhi Zhu, Bei Wu, Hanzhang Xu, Jing Wang, Juan Li

**Affiliations:** Naval Military Medical University, Shanghai, Shanghai, China (People's Republic); Naval Military Medical University, Shanghai, Shanghai, China (People's Republic); New York University, New York, New York, United States; Duke University, Durham, North Carolina, United States; University of North Carolina at Chapel Hill, Chapel Hill, North Carolina, United States; Huashan Hospital affiliated to Fudan University, Shanghai, Shanghai, China (People's Republic)

## Abstract

This study aims to investigate the equivalence between Cambridge Neuropsychological Test Automated Battery (CANTAB) and Montreal Cognitive Assessment, Changsha version (MoCA-CS) as cognition measures for Chinese stroke survivors. Sixteen stroke survivors were recruited from stroke center in Shanghai, China. Participants completed Paired Associates Learning (PAL) task in CANTAB, MoCA-CS and questionnaire about demo-social characteristics. Pearson correlation and hierarchical regression were used for equivalence analysis. CANTAB-PAL task performances（PAL First Attempt Memory Score, PALFAMS, PAL Total Attempts 2 Patterns, PALTA2）were significantly associated with score of MoCA-CS and delayed recall (β=0.528, p=0.014; β=0.198, p=0.043; β= -3.885，p=0.017; β= -1.600,p=0.026, respectively）after controlling for age, gender and education. The results suggest CANTAB-PAL is a reliable tool for measuring Chinese stroke survivors’ cognitive function. Future studies are needed to establish the equivalence of CANTAB in multi-tasks in larger sample of Chinese stroke survivors.

